# Advances in artificial intelligence empowering early-stage human health: Current landscape and future directions

**DOI:** 10.1016/j.xinn.2025.101222

**Published:** 2025-12-03

**Authors:** Nana Zheng, Xinyu Zhang, Chao Li, Forrest Tin Wai Cheung, Xiaoqing Hu, Jihui Zhang, Hongliang Feng

**Affiliations:** 1 Center for Sleep and Circadian Medicine, The Affiliated Brain Hospital, Guangzhou Medical University, Guangzhou 510370, China; 2 Guangdong Engineering Technology Research Center for Translational Medicine of Mental Disorders, Guangzhou 510370, China; 3 Center for Reproductive Medicine, Peking University Third Hospital, Beijing 100191, China; 4 College of Computer and Information Engineering, Tianjin Normal University, Tianjin 300387, China; 5 Sir Jules Thorn Sleep and Circadian Neuroscience Institute, Nuffield Department of Clinical Neurosciences, University of Oxford, Oxford OX1 3QU, UK; 6 Department of Psychology, The University of Hong Kong, Pokfulam, Hong Kong SAR 999077, China; 7 Li Chiu Kong Family Sleep Assessment Unit, Department of Psychiatry, Faculty of Medicine, The Chinese University of Hong Kong, Hong Kong SAR 999077, China

## Main text

**Figure 1:**
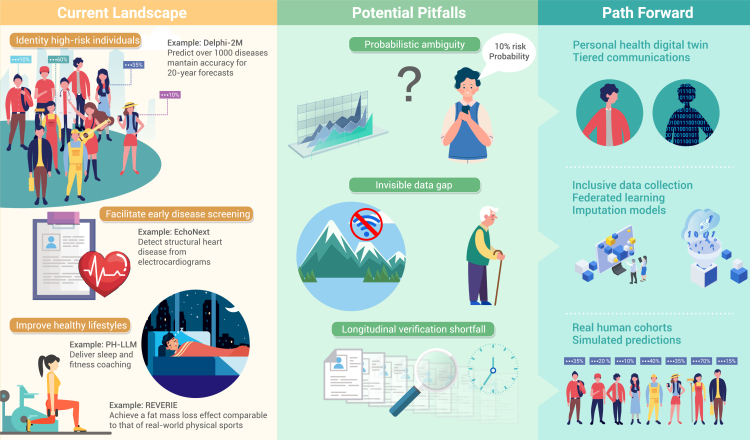
The application landscape, inherent challenges, and future directions of artificial intelligence in early-stage human health promotion

While healthcare providers have used computer-aided programs since the 1950s, artificial intelligence (AI) in health promotion has only recently flourished, driven by advances in large language models (LLMs), discriminative machine learning, and multimodal foundation models. Fueled by growing data and computing power, AI now excels in drug development, diagnostic support, and AI-assisted surgery. The ancient Eastern concept of “preventing disease before it occurs” and modern preventive medicine both aim to safeguard health through early proactive intervention. Amid global aging and rising incidence of complex chronic diseases, which are often hard to treat once developed, early health promotion is increasingly crucial. Studies suggest AI tools hold great promise for reshaping early-stage health promotion, yet this field faces distinctive challenges, and its future development pathways warrant in-depth considerations (Figure 1).

## Current landscape

Early precision prevention relies on identifying high-risk individuals. A recent study shows AI’s remarkable potential in this area.^1^ Trained on data from 402,799 UK Biobank participants (including demographics, lifestyle, and 1,256 diseases) and validated on the 1.93 million Danish registry records, the Delphi-2M predicts over 1,000 diseases and mortality with mean area under the receiver operating characteristic curve (AUC) of 0.69 (UK) and 0.67 (Danish) over 20 years.^1^ However, the model has limitations, including healthy volunteer bias and the impact of diverse data sources and missingness patterns on predictions and variable performance across ancestry and deprivation groups. These issues highlight the need for caution with heterogeneous healthcare datasets and position the model as a supplement, not a replacement, for clinical judgment.

Early screening is key to preventing multisystem diseases from becoming chronic. AI has advanced significantly in addressing long-standing gaps in early disease screening. For example, using 12-lead electrocardiograms and basic demographics, EchoNext identifies a wide range of structural heart diseases with an AUC of 85% in internal validation and 78%–80% in external cohorts, outperforming 13 board-certified cardiologists (64% accuracy).^2^ Additionally, a “silent deployment” of EchoNext achieved a 74% positive predictive value for structural heart disease in individuals without prior echocardiograms,^2^ addressing the field’s heavy reliance on this method. However, the small sample size of the prospective trial is a key limitation, and larger studies are needed to confirm benefits in routine care.

Maintaining a healthy lifestyle is a well-documented way to prevent diseases and prolong life. Promising evidence suggests a paradigm shift from passive data collection to active health guidance. For instance, personal health LLM (PH-LLM) outperformed human experts on sleep medicine (79% vs. 76%, *n* = 629) and fitness (88% vs. 71%, *n* = 99) multiple-choice exams sourced from BoardVitals and aligned with NSCA-CSCS standards, respectively.^3^ PH-LLM also performed similarly to experts in an evaluation involving 350 real-world case studies.^3^ However, exam scores are only surrogate measures reflecting knowledge proficiency rather than long-term health impact. Another limitation is sample representativeness bias (e.g., fitness cases enriched with males aged 30–59 years), weakening the generalizability.

AI-based virtual reality (VR) exercise systems have shown promise in promoting healthy behaviors.^4^ For example, REVERIE, an AI-based VR system using deep reinforcement learning to train transformer-based virtual coaches, effectively reduced the primary outcome of fat mass (mean −4.28 kg, vs. control) in adolescents with excess body weight.^4^ This effect did not differ significantly from real-world physical sports. However, the study did not assess the system's impact on cardiorespiratory fitness and lacked data from long-term follow-up data.

## Potential pitfalls

Beyond the common medical AI challenges (e.g., external validity, data integration) noted elsewhere,^5^ AI applications in early-stage health promotion are more likely to be direct-to-consumer, thereby facing a series of unique challenges.

Even ignoring current model accuracy limit and assuming precise risks, the public may still be misled by the “probabilistic ambiguity” inherent in long-term health predictions. For instance, AI predicting an individual’s disease risk over the next 10–20 years (e.g., a 10% probability of colorectal cancer), confronts a public that often lacks probabilistic reasoning, tending to mistake “probability” for “deterministic outcome”: some may overreact to a “10% risk” (e.g., frequent colonoscopies), while others may neglect prevention due to a perceived “90% safety” (e.g., forgoing necessary screenings).

“invisible data gaps” among vulnerable populations may exacerbate inequalities in health services. Since AI in early-stage health promotion relies on daily data (e.g., from wearables), groups like elderly, low-income groups, and remote residents, who often lack such devices, are severely underrepresented in training datasets. For instance, Delphi-2M was constructed using UK Biobank data, which are known to overrepresent healthier individuals from less deprived areas.^1^ Thus, these gaps may cause AI to fail in addressing the health needs of vulnerable populations.

Current AI tools for early health promotion focus on aligning predictions with established knowledge and validating short-term effects. However, even high-performing models may underdeliver in practice. For instance, PH-LLM outperformed human experts in theoretical exams but showed only comparable or sometimes inferior performance in real-world case analyses.^3^ The true impact of early health behavior interventions requires years or even decades of observation, and the long-term health effects of AI-assisted interventions remain unclear. For example, while exercise is known to reduce depression risk, whether novel modalities such as REVERIE^4^ can still yield comparable benefits requires further research.

## Path forward

Future breakthroughs in personal health digital twins may help address public misinterpretation of health prediction probabilities. Instead of being limited to analyzing isolated physical examination data, AI will integrate an individual’s genome, proteome, continuous physiological data, and even behavioral data to create an accurate and dynamic digital model. For interpretation, a visual twin can convert abstract probabilities into tangible health trajectories (e.g., color-coded timelines showing colorectal cancer risk changes over decades under different lifestyles). A tiered strategy could provide interactive tools to build probabilistic thinking, supplemented by one-on-one counseling for high-risk groups. Developers should also embed risk interpretation modules, generating user-friendly reports and linking to medical institutions to form a “prediction-interpretation-intervention” loop.

For next-generation AI tools, governments, developers, and other stakeholders should explore ways to ensure the inclusion of vulnerable populations’ data. Governments could provide data collection assistance through primary care, while developers adopt tailored methods for these groups. In addition, federated learning paired with advanced imputation models should be deployed: federated learning integrates data across regions to prevent silos; for those with insufficient data, imputation models can simulate health needs based on group traits, bridging data gaps. Meanwhile, regulators should require AI health tools entering the market to disclose their populations’ limitations, creating an incentive for developers.

AI may become a 24/7 “AI health coach,” driving innovation in intervention methods and highlighting the importance of life cycle monitoring. Long-term population observation remains a robust way to evaluate these coaches’ sustained performance. Regulatory authorities should consider integrating such monitoring and assessment into the approval criteria. However, long-term studies face time and resource constraints. Digital twin technology can expedite the verification: by constructing individual health twins from short-term cohort data to simulate the prolonged intervention impacts, and then calibrating the simulation results with actual mid-term cohort data, it can significantly shorten the validation cycle.

Guidelines or consensus are urgently needed to steer stakeholders in advancing early health promotion AI tools. No prior guidelines or consensus have adequately addressed this field’s unique attributes, such as direct public access and fragmented scenarios. While guidelines frameworks like FUTURE-AI (https://future-ai.eu/) offer a reference, they require adaptation for early health promotion. For instance, regarding explainability, public-facing expressions should be optimized, requiring AI to translate professional advice into actionable daily steps rather than clinical jargon—to advance trustworthy, deployable AI tools.

## Funding and acknowledgments

H.F. was supported by the National Science and Technology Innovation 2030 of China-Major Projects (2022ZD0214100), the 10.13039/501100001809National Natural Science Foundation of China (82571696), Guangzhou Municipal School (College)-Enterprise Joint Funding Project (2024A03J0214), and Guangzhou Science and Technology Plan Project (2025A03J3929). This work was supported by Guangzhou Key Clinical Specialty (Clinical Medical Research Institute). The funders had no role in study design, data collection and analysis, decision to publish, or preparation of the manuscript.

## Declaration of interests

The authors declare no competing interests.
